# Age associated changes in behavioral and memory functions in genetically heterogeneous mice UM‐HET3

**DOI:** 10.1002/alz.095615

**Published:** 2025-01-09

**Authors:** Harpreet Kaur, Bretton Badenoch, Peter C. Reifsnyder, Kristen MS O'Connell, Richard A. Miller, David E. Harrison, Catherine C. Kaczorowski

**Affiliations:** ^1^ University of Michigan, Ann Arbor, MI USA; ^2^ The Jackson Laboratory, Bar Harbor, ME USA

## Abstract

**Background:**

Previous studies have documented age‐related changes in behavior and cognitive functions and investigated the molecular changes in aging brain using inbred mouse strains such as C57BL/6, BALB/c etc. In this study using a genetically heterogenous mouse population (UM‐HET3) we investigated age‐related changes in motor and memory functions and their association with blood cell measures.

**Method:**

Both male and female UM‐HET3 mice at age of 11 months (middle‐aged) and 25 months (old) were used in this study. Complete blood count, grip‐strength, Rotarod, visual acuity, startle response, voluntary wheel running activity and contextual fear conditioning assays were performed on these mice. Further, a correlation analysis was performed on blood cells and contextual fear memory (CFM) to identify signatures associated with age related cognitive decline.

**Result:**

Our results showed significantly lower body temperature, decrease in hemoglobin, changes in blood related parameters (including RBC, hematocrit, platelets, MPV, WBC), reduced visual acuity, reduced grip strength (not normalized by BW) and impaired memory functions in older mice when compared to middle‐aged mice. We found no correlation between blood cells and CFM in middle‐aged mice, but the percentage of neutrophils (R = 0.794, p = 0.009) and lymphocytes (R = ‐0.734, p = 0.022) were found to be associated with CFM in older male mice. A reduced startle reactivity was also observed in older male and female mice which is likely to be the result of impaired hearing, although these mice were not tested for hearing loss. No statistical significance was observed in Rotarod performance and in wheel running activity when middle aged mice compared to old UM‐HET3 mice.

**Conclusion:**

Our study showed that UM‐HET3 mice exhibit age related impairment in sensory and memory functions as compared to middle aged mice. In older mice, reduced memory correlates with increased percentage of neutrophils and decreased lymphocytes in blood, suggesting a possible role of white blood cells in age‐related memory decline in UM‐HET3 mice. However, further studies are needed to understand the mechanisms driving age‐related decline in cognition with focus on changes in immune cells for developing therapies to slow down the rate of cognitive decline in normal aging as well as neurological disorders such as Alzheimer’s disease.

